# Effect of scar and pacing location on repolarization in a porcine myocardial infarction model

**DOI:** 10.1016/j.hroo.2022.01.008

**Published:** 2022-01-26

**Authors:** Mark K. Elliott, Caroline Mendonca Costa, John Whitaker, Philip Gemmell, Vishal S. Mehta, Baldeep S. Sidhu, Justin Gould, Steven E. Williams, Mark O’Neill, Reza Razavi, Steven Niederer, Martin J. Bishop, Christopher A. Rinaldi

**Affiliations:** ∗ School of Biomedical Engineering and Imaging Sciences, King’s College London, London, United Kingdom; † Department of Cardiology, Guy’s and St Thomas’ NHS Foundation Trust, London, United Kingdom

**Keywords:** Cardiac magnetic resonance imaging, Electroanatomic mapping, Myocardial scar, Repolarization, Ventricular arrhythmia

## Abstract

**Background:**

The effect of chronic ischemic scar on repolarization is unclear, with conflicting results from human and animal studies. An improved understanding of electrical remodeling within scar and border zone tissue may enhance substrate-guided ablation techniques for treatment of ventricular tachycardia. Computational modeling studies have suggested increased dispersion of repolarization during epicardial, but not endocardial, left ventricular pacing, in close proximity to scar. However, the effect of endocardial pacing near scar *in vivo* is unknown.

**Objective:**

The purpose of this study was to investigate the effect of scar and pacing location on local repolarization in a porcine myocardial infarction model.

**Methods:**

Six model pigs underwent late gadolinium enhancement cardiac magnetic resonance (LGE-CMR) imaging followed by electroanatomic mapping of the left ventricular endocardium. LGE-CMR images were registered to the anatomic shell and scar defined by LGE. Activation recovery intervals (ARIs), a surrogate for action potential duration, and local ARI gradients were calculated from unipolar electrograms within areas of late gadolinium enhancement (aLGE) and healthy myocardium.

**Results:**

There was no significant difference between aLGE and healthy myocardium in mean ARI (304.20 ± 19.44 ms vs 300.59 ± 19.22 ms; *P* = .43), ARI heterogeneity (23.32 ± 11.43 ms vs 24.85 ± 12.99 ms; *P* = .54), or ARI gradients (6.18 ± 2.09 vs 5.66 ± 2.32 ms/mm; *P* = .39). Endocardial pacing distance from scar did not affect ARI gradients.

**Conclusion:**

Our findings suggest that changes in ARI are not an intrinsic property of surviving myocytes within scar, and endocardial pacing close to scar does not affect local repolarization.

**Key Findings**

▪ There were no detectable differences in mean activation recovery interval (ARI), ARI heterogeneity, or local ARI gradients between areas of late gadolinium enhancement (aLGE) and healthy myocardium.
▪ There were no significant differences in mean ARI, ARI heterogeneity, or local ARI gradients in either aLGE or healthy myocardium when right ventricular (RV) and left ventricular (LV) pacing were compared.
▪ There was no significant difference in ARI gradients within aLGE when pacing in close proximity to vs distant from scar.
▪ When repolarization gradients were calculated instead of ARI, there were no differences observed between aLGE and healthy myocardium or between RV and LV pacing, and there was similarly no effect of pacing distance from scar.

## Introduction

Ischemic heart disease is the leading cause of premature death worldwide.^1^ Over recent decades, improvements in diagnosis, drug therapy, and revascularization have led to increased survival after myocardial infarction,^2^^,^^3^ resulting in an increasing population of patients living with chronic infarcts. Ventricular arrhythmias cause significant morbidity and mortality in patients with previous myocardial infarction.^4^ Catheter ablation is an important clinical tool in the management of patients with scar-related ventricular tachycardia (VT), reducing hospitalization, VT storm, and implantable cardioverter-defibrillator shocks.^5^ However, success rates are variable, with 1-year recurrence rates up to 50%,^6^ thus highlighting the need for improved technologies that more accurately identify the myocardial substrate sustaining the VT. A significant proportion of patients undergoing ablation have hemodynamically unstable VT, thus limiting the use of techniques such as activation mapping and entrainment.^7^ In such patients, a substrate-guided approach is often the only option, in which potentially arrhythmogenic tissue is mapped in either sinus or paced rhythm. Whereas current substrate mapping techniques rely on targeting tissue depending on voltage-based definitions of scar or on abnormal late potentials, outcomes potentially could be improved by incorporating an assessment of electrical remodeling. Indeed, strategies incorporating conduction velocity and activation time deceleration have been described.^8^^,^^9^ Furthermore, a high reentry vulnerability index, which incorporates local activation and repolarization times, has been spatially correlated with VT sites of origin.^10^^,^^11^

During infarct healing, cardiac myocytes are replaced by fibrous tissue, with the peripheral border zone of the scar characterized by surviving myocytes surrounded by fibrous tissue. Altered myofiber architecture results in conduction slowing, which is further exacerbated by gap junction remodeling, resulting in abnormal cell-to-cell coupling.^12^ Electrical remodeling within the border zone may also alter the action potential duration, resulting in local dispersion of repolarization, which has been associated with arrhythmogenesis.^13^ However, the effect of electrical remodeling in the border zone on repolarization is poorly understood. Although action potential duration shortening has been demonstrated in the border zone during the subacute "healing" phase of infarction in human studies and animal models, changes in action potential duration during the chronic "healed" phase are less well established, with studies showing conflicting results.^14^^,^^15^ Obtaining a more detailed understanding of action potential duration heterogeneity and its link to tissue type and stage of infarction will provide key insights into the mechanisms of arrhythmogenesis in patients with previous myocardial infarction. This may have clinical implications in the improvement of substrate mapping techniques for VT ablation and the development of new antiarrhythmic drugs and other novel therapies for myocardial scar, such as stem cells and myocardial grafts.^16^

Given that local, rather than global, dispersion of repolarization likely contributes to the substrate required for reentry, calculation of local spatial gradients of repolarization may help in identifying arrhythmogenic tissue. Recent modeling studies by our group have demonstrated higher local repolarization gradients during epicardial, but not endocardial, pacing in close proximity to scar, which may have important clinical implications for the delivery of cardiac resynchronization therapy (CRT) in patients with ischemic cardiomyopathy.^17^^,^^18^ The effect of endocardial pacing near scar *in vivo* is unknown.

We hypothesized that surviving myocytes within scar would have altered action potential duration in comparison to healthy myocardium, thus creating high dispersion repolarization and the substrate for arrhythmia. We also hypothesized that endocardial pacing in proximity to scar may affect repolarization. We performed late gadolinium enhancement cardiac magnetic resonance (LGE-CMR) imaging and comprehensive electroanatomic mapping studies on a cohort of porcine models of myocardial infarction, during a variety of pacing protocols. Unipolar electrograms were directly analyzed to calculate activation recovery intervals (ARIs), an established surrogate for action potential duration.^19^ We investigated the differences in ARI and local repolarization gradients between areas of late gadolinium enhancement (aLGE) and healthy myocardium during right ventricular (RV) and left ventricular (LV) endocardial pacing, and the effect of pacing distance from scar.

## Methods

### Animal model

Six domestic white swine underwent a 180-minute balloon occlusion of the left anterior descending artery to create an ischemia-reperfusion myocardial infarction model as previously described.^20^, ^21^, ^22^ The protocol was approved by the Institutional Animal Care and Use Committee, conformed to the position of the American Heart Association on Research Animal Use, and was performed in compliance with the ARRIVE guidelines. Pigs were treated with amiodarone 800 mg twice daily for 4 days to reduce the risk of death from ventricular arrhythmia. Anteroseptal ischemia-reperfusion myocardial infarction was created as previously described.^20^ Oral amiodarone was continued for an additional 4–5 days at a dose of 800 mg twice daily and then stopped. An additional pig underwent a sham balloon occlusion procedure and was used as a control.

### Cardiac magnetic resonance imaging data acquisition

LGE-CMR imaging was acquired a median of 53.5 (range 50–57) days postinfarct. All imaging was performed on 1.5-T scanner (MAGNETOM Aera, Siemens Healthineers, Erlangen, Germany) with an 18-channel body matrix coil and a 32-channel spine coil. Isotropic navigator-gated electrocardiogram-triggered 3-dimensional inversion recovery sequence was acquired (balanced steady-state free precession readout; coronal orientation; linear k-space reordering; TE/TR/α: 1.58 ms/3.6 ms/90°; gating window 7mm; parallel imaging using GRAPPA with acceleration factor of 2; resolution 1.2 × 1.2 × 1.2 mm^3^; field of view 400 × 257 × 96 mm^3^) with full ventricular coverage.^21^^,^^22^

### Electrophysiological study

Electrophysiological study was performed a median of 63 (range 56–64) days postinfarction using a Precision™ electroanatomic mapping system (Abbott, Minnesota, MN) with pacing stimulator under general anesthesia. Hemodynamic support was provided prophylactically at the start of the procedure using venous-arterial extracorporeal membrane oxygenation delivered using a Maquet Cardiohelp machine (Maquet Getinge Group, Rastatt, Germany). Unfractionated heparin was given as a 100 U/kg bolus intravenously, followed by 40–50 U/kg/h infusion to maintain an activated clotting time of 180–250 seconds. A decapolar catheter (Livewire, Abbott) and a pentapolar catheter (Boston Scientific, Marlborough, MA) were placed in the RV apex and coronary sinus, respectively, via femoral venous access. A sensor-enabled Abbott FlexAbility™ ablation catheter was placed in the LV cavity via femoral arterial access and retrograde aortic approach and was used to create endocardial geometry and for LV endocardial pacing. LV endocardial activation maps were acquired using a multipolar mapping catheter (HD Grid™ or LiveWire™ Duo-Deca; Abbott, Chicago, IL) advanced through an Agilis sheath (Abbott, Chicago, IL) via the aorta, while pacing from the RV apex at 500 ms and 300 ms cycle lengths. In 4 pigs, further activation maps were acquired during LV endocardial pacing at 500 and 300 ms. In all pigs, an adapted Wellen’s VT stimulation protocol was performed for VT induction, with up to 4 extrastimuli.^23^ LV geometry and acquired electrograms for each map were exported from Precision and imported into MatLab (MathWorks, Natick, MA) using OpenEP software.^24^

### Electrogram analysis

**Figure 1:**
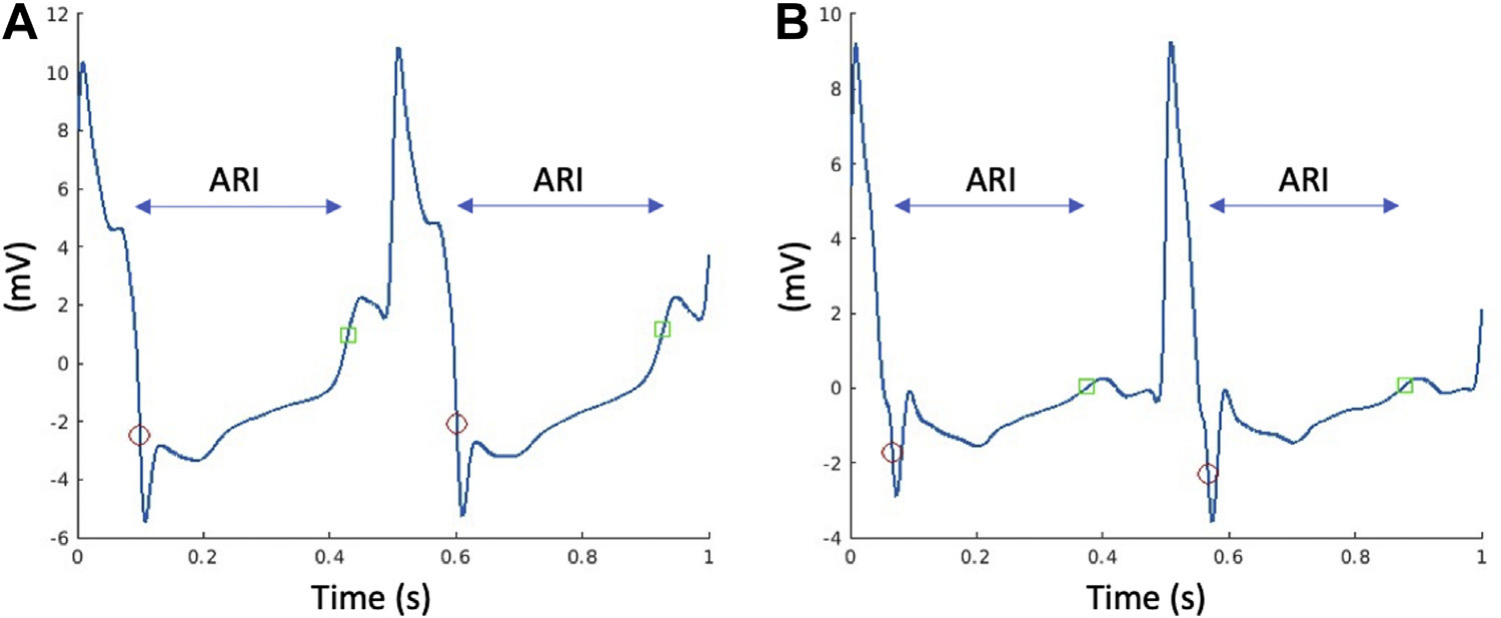
Example of unipolar endocardial electrograms with calculated activation recovery intervals (ARIs). **A:** Electrogram from healthy myocardium. **B:** Electrogram from an area of late gadolinium enhancement. *Red circles* indicate local activation times. *Green squares* indicate repolarization times.

One-second recordings of endocardial electrograms were exported from all mapped points together with their corresponding triangulated shell. Unipolar electrograms were low-pass filtered at 80 Hz to remove high-frequency noise.^25^ Standard bipolar signals were created from neighboring poles of the multipolar mapping catheters. Local activation time was calculated from a bespoke algorithm utilizing both unipolar and bipolar signals to more effectively eliminate spurious activations. Specifically, the initial estimate for activation time from the Precision software was used to define an initial search window. Within this window, the maximal negative derivative (dV/dt_max_) of the QRS segment^19^ of the unipolar electrogram was located and used with the timing of the pacing stimulus to define the local activation time. Local repolarization time was calculated using the traditional Wyatt method as previously described.^19^^,^^25^ A search window initially was defined based on the local activation time, which was shortened as required to avoid conflict with depolarization or repolarization from the preceding or following QRS complex. T-wave peak was found within that window. If no maximum peak was found within the window, the T wave was assumed to be negative, and the negative peak was used. If the T wave was positive, repolarization time was determined as the point of maximum derivative (dV/dt_max_) before the peak. If the T wave was negative, repolarization time was determined as the point of dV/dt_max_ following the peak. Example unipolar electrograms from healthy myocardium and areas of LGE with annotated activation and repolarization times are shown in Figure 1. ARI was defined as the time interval between the activation time and repolarization time and was computed for all beats within the 1-second exported segment, constituting 2 or 3 full complexes for the 500 ms and 300 ms pacing protocols, respectively. An additional strategy was applied to remove possible noise in the measurements of ARIs. ARI data were excluded from all sites in which the ARI calculated from the second exported beat differed by >10% from the first exported beat, which removed between a mean of 26% and 38% of recorded data for pacing at 300 and 500 ms, respectively. This 10% threshold is approximately 10-fold greater than the physiological beat-to-beat variability in ARI, which has previously been described.^26^ After filtering, the difference in mean ARI between the 2 exported beats was 0.7 ms (0.3%).

### Image analysis

For each pig, the high-resolution LGE-CMR images were segmented to identify LV myocardium. Tissue within the myocardium was automatically classified as scar, border zone, or healthy tissue based on the signal intensity of the CMR imaging as previously described.^22^ The LV geometry was imported into the electroanatomic mapping system, and the registration tool within the system was used to register the LV geometry to the activation mapping data. Activation mapping data and LV geometry were then exported from the electroanatomic mapping system within the same image space, and further processing was performed offline. The LV geometry material tags (scar, border zone, and healthy) and scar distance values were interpolated onto the electroanatomic shells using an iterative nearest neighbor method, implemented within MESHTOOL.^27^

**Figure 2:**
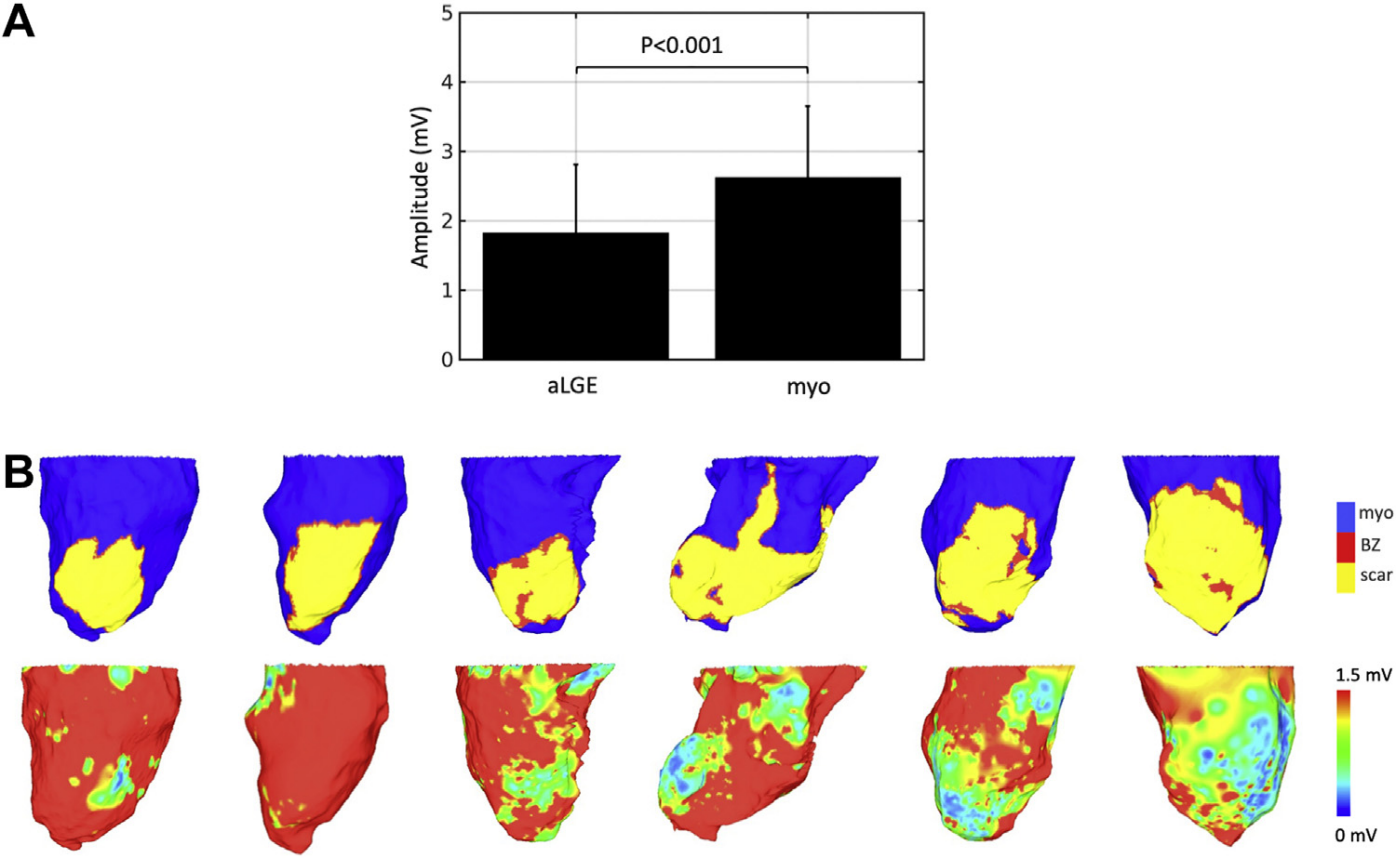
Comparison of late gadolinium enhancement cardiac magnetic resonance (LGE-CMR) and bipolar voltage maps for scar detection. **A:** Bipolar voltage amplitude in areas of late gadolinium enhancement (aLGE) and healthy myocardium (myo) determined by cardiac magnetic resonance. **B:** Maps of LGE-CMR derived tissue type **(top row)** with corresponding bipolar voltage maps **(bottom row)** for each pig. BZ = border zone.

To address potential registration errors between the imaging data and the activation mapping data, a further step was introduced to filter points within close proximity to the interface between healthy tissue and scar, where the potential for misclassification due to registration errors was highest. To accomplish this, an estimate of the geodesic distance of each node from the scar–healthy interface was calculated using LGE-based computational models and eikonal simulations, as described previously.^17^ Using the mapped material tags and scar distance, regions of scar (including border zone) and healthy myocardium were defined according to the material tag and any points within 2 mm of the scar–healthy tissue interface were excluded. The areas defined as border zone by LGE signal intensity were small (Figure 2B), so the acquired electrograms and calculated ARIs from regions labeled as both border zone and scar were combined and represent signals from surviving myocytes within areas of CMR-defined LGE. This tissue is referred to as aLGE during analysis.

### ARI analysis

The ARI of the first full complex (beat) of each electrogram was interpolated onto the corresponding shell using a global Sheppard interpolation method, implemented within the freely available MESHTOOL.^27^ Local spatial gradients of ARI were computed on the triangulated shells, whereby the ARI gradient at each point reflects the local rate of change in ARI relative to neighboring points directly connected through an element edge. Mean ARI and ARI gradient within aLGE and healthy myocardium were computed for each pig and pacing protocol. In addition, ARI heterogeneity was computed as the standard deviation of ARI values within aLGE and healthy myocardium. The same process was performed for repolarization time to create repolarization time gradients for both aLGE and healthy myocardium. The interpolation process and ARI analysis were repeated using the ARI of the second exported complex of each electrogram and the mean ARI of both complexes. The results reported in the main study are for the first exported complex. The results for the second complex and average of the 2 complexes are provided in Supplemental Tables 1 and 2, respectively.

### Statistical analysis

The mean and standard devation of metrics for aLGE vs myocardium were compared using a paired Student *t* test. A repeated measures analysis of variance (ANOVA) was used when comparing RV vs LV endocardial pacing, for the four pigs who underwent both protocols. An unbalanced ANOVA was used for unequal sized groups. Correlation between groups was assessed by computing the Pearson correlation coefficient followed by a *t* test. *P* <.05 was considered significant. Outliers were defined as values above or below the 10th and 90th percentiles for a given data group and were excluded from analysis.

## Results

Six ischemia-reperfusion infarction pig models were successfully created and underwent LGE-CMR and electrophysiological study. One sham control pig also underwent electrophysiological study. VT was successfully induced in all the infarct pigs, and all had spontaneous nonsustained VT during the electrophysiological procedure. The sham control pig did not have any nonsustained VT or inducible VT.

### Comparison of LGE-defined scar and bipolar voltage amplitude

aLGE had significantly lower bipolar voltage compared to healthy myocardium (1.83 ± 0.97 mV vs 2.62 ± 1.03 mV; *P* <.001) (Figure 2A). However, there was discrepancy in the visual correlation between aLGE and bipolar voltage scar maps (Figure 2B), with areas of low voltage often seen around the base of the heart where LGE was not present. This may be related to low-quality electrograms secondary to poor contact in these regions. For this reason, scar was defined according to LGE signal intensity as described in the Methods section. Mean bipolar voltage in the sham control pig was 2.73 ± 1.27 mV, which was comparable to the voltage in healthy myocardium of the infarct pigs (Supplemental Figure 1).

### ARIs

**Figure 3:**
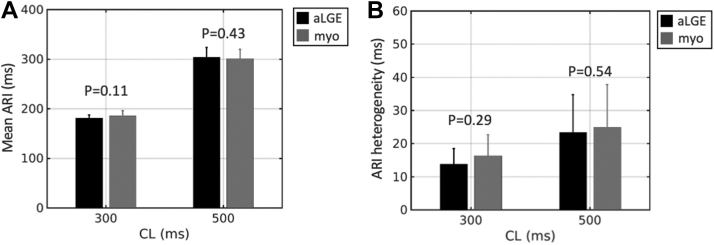
Activation recovery interval (ARI) by tissue type. Mean ARI **(A)** and ARI heterogeneity **(B)** in areas of late gadolinium enhancement (aLGE) vs healthy myocardium (myo) at 300 ms and 500 ms pacing cycle lengths (CL).

**Figure 4:**
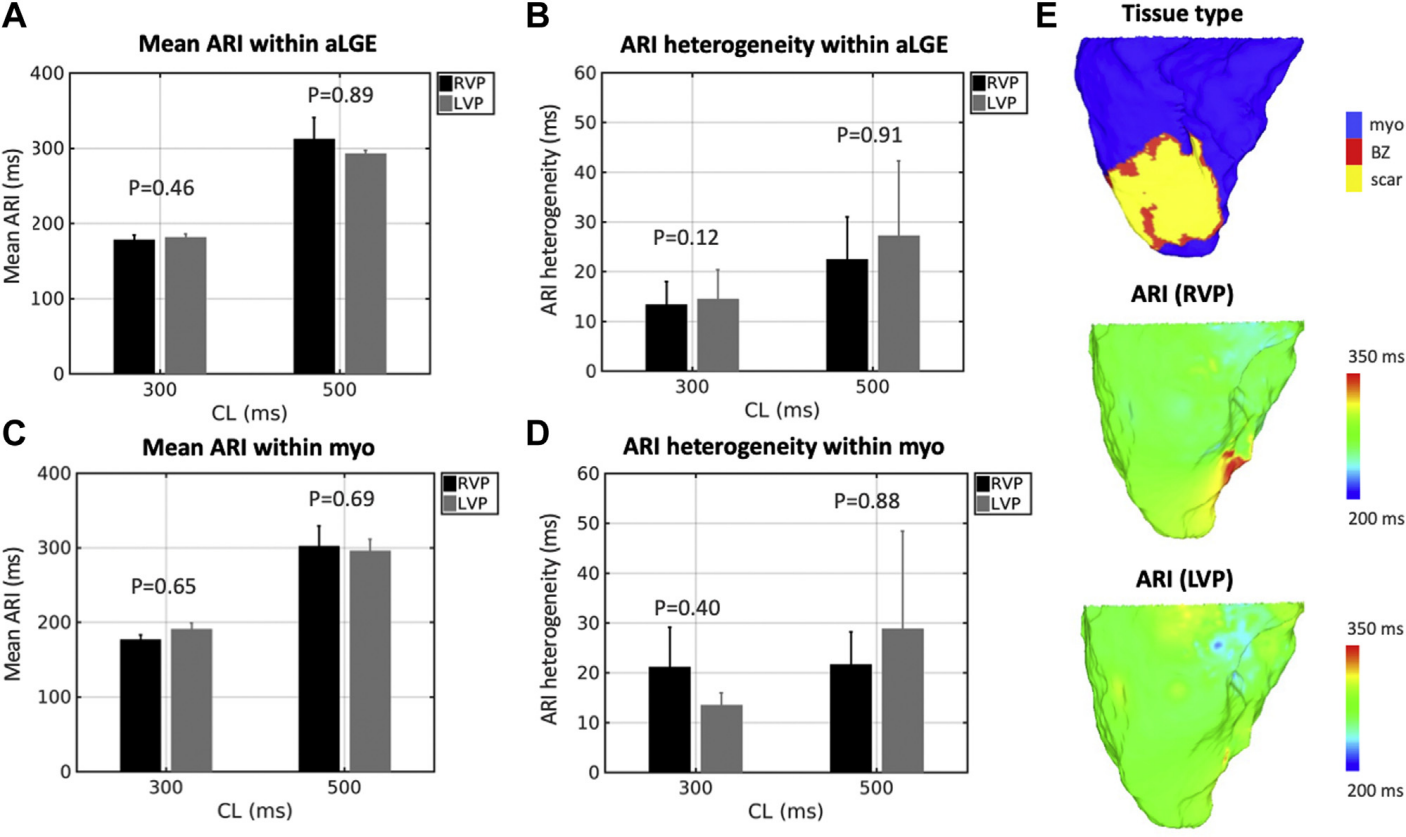
Activation recovery interval (ARI) by pacing location. Mean ARI (**A**) and ARI heterogeneity (**B**) in areas of late gadolinium enhancement (aLGE) during right ventricular pacing (RVP) and left ventricular endocardial pacing (LVP). Mean ARI (**C**) and ARI heterogeneity (**D**) in areas of healthy myocardium (myo) during RVP and LVP. (**E**) Example maps of tissue type and ARI during RVP and LVP at 500-ms pacing cycle length (CL). BZ = border zone.

ARI was measured at 85,927 points (19% aLGE), with a mean of 4296 points per pig. Mean ARI in aLGE and healthy myocardium was not significantly different at either 300 ms (181.36 ± 5.91 ms vs 185.93 ± 10.02 ms; *P* = .11) or 500 ms (304.20 ± 19.44 ms vs 300.59 ± 19.22 ms; *P* = .43) pacing cycle length (PCL) (Figure 3A). ARI heterogeneity was also similar between tissue types (300 ms PCL: 13.77 ± 4.71 ms vs 16.32 ± 6.38 ms; *P* = .29; 500 ms PCL: 23.32 ± 11.43 ms vs 24.85 ± 12.99 ms; *P* = .54) (Figure 3B). When comparing RV pacing vs LV endocardial pacing, in aLGE there was no difference in mean ARI (300 ms PCL: 178.21 ± 6.33 ms vs 181.48 ± 4.49 ms; *P* = .46; 500 ms PCL: 312.31 ± 28.51 ms vs 293.19 ± 4.04 ms; *P* = .89) (Figure 4A) or ARI heterogeneity (300 ms PCL: 13.38 ± 4.62 ms vs 14.49 ± 5.91 ms; *P* = .12; 500 ms PCL: 22.46 ± 8.56 ms vs 27.25 ± 15.01 ms; *P* = .91) (Figure 4B). For healthy myocardium, mean ARI (300 ms PCL: 176.81 ± 6.20 ms vs 191.04 ± 7.88 ms; *P* = .65; 500 ms PCL: 301.90 ± 27.34 ms vs 296.02 ± 15.37 ms; *P* = .69) and ARI heterogeneity (300 ms PCL: 21.17 ± 7.94 ms vs 13.53 ± 2.46 ms; *P* = .40; 500 ms PCL: 21.68 ± 6.50 ms vs 28.80 ± 19.60 ms; *P* = .88) were similar for RV pacing and LV endocardial pacing, respectively (Figures 4C and 4D). In agreement with the numerical analysis, ARI maps did not show a visual correlation between ARI and tissue type (Figure 4E). For comparison, in the sham control pig, mean ARI was 245.61 ms, and ARI heterogeneity was 15.57 ms during RV pacing at 500 ms PCL (Supplemental Figure 1). The findings were unchanged when the analysis was performed using the second exported beat (Supplemental Table 1) and the average of both exported beats (Supplemental Table 2).

### Local ARI gradients

**Figure 5:**
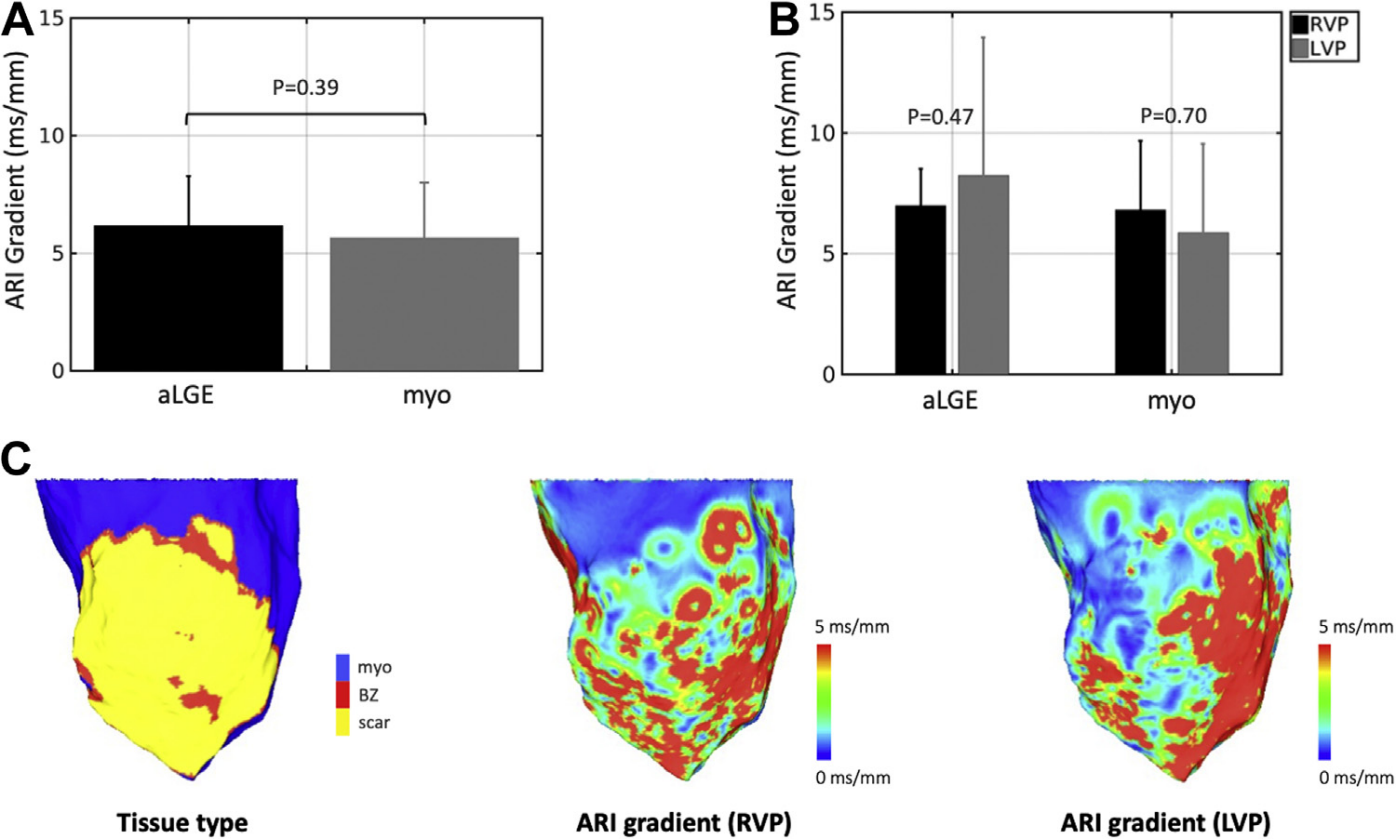
Activation recovery interval (ARI) gradients. **A:** Mean ARI gradient in areas of late gadolinium enhancement (aLGE) vs healthy myocardium (myo). **B:** Mean ARI gradient within aLGE and myo during right ventricular pacing (RVP) and left ventricular endocardial pacing (LVP). **C:** Example maps of tissue type and ARI gradients during RVP and LVP. BZ = border zone.

For all PCLs analyzed together, there was no significant difference in ARI gradients between aLGE and healthy myocardium (6.18 ± 2.09 ms/mm vs 5.66 ± 2.32 ms/mm; *P* = .39) (Figure 5A). Comparing RV pacing with LV pacing, there also was no difference in ARI gradients in either aLGE (7.0 ± 1.51 ms/mm vs 8.24 ± 5.72 ms/mm; *P* = .47) or healthy myocardium (6.82 ± 2.84 ms/mm vs 5.87 ± 3.67 ms/mm; *P* = .70) (Figure 5B). ARI gradient maps demonstrated no visual correlation between ARI gradients and tissue type, in agreement with the numerical data (Figure 5C). There also was no difference in local repolarization time gradients between aLGE and healthy myocardium, or between RV pacing and LV pacing in either aLGE or healthy myocardium (Supplemental Figure 2). For comparison, mean ARI gradients were 5.71 ± 6.74 mm/ms in the sham control pig during RV pacing at 500 ms (Supplemental Figure 1).

### Effect of pacing distance from scar on ARI gradients

**Figure 6:**
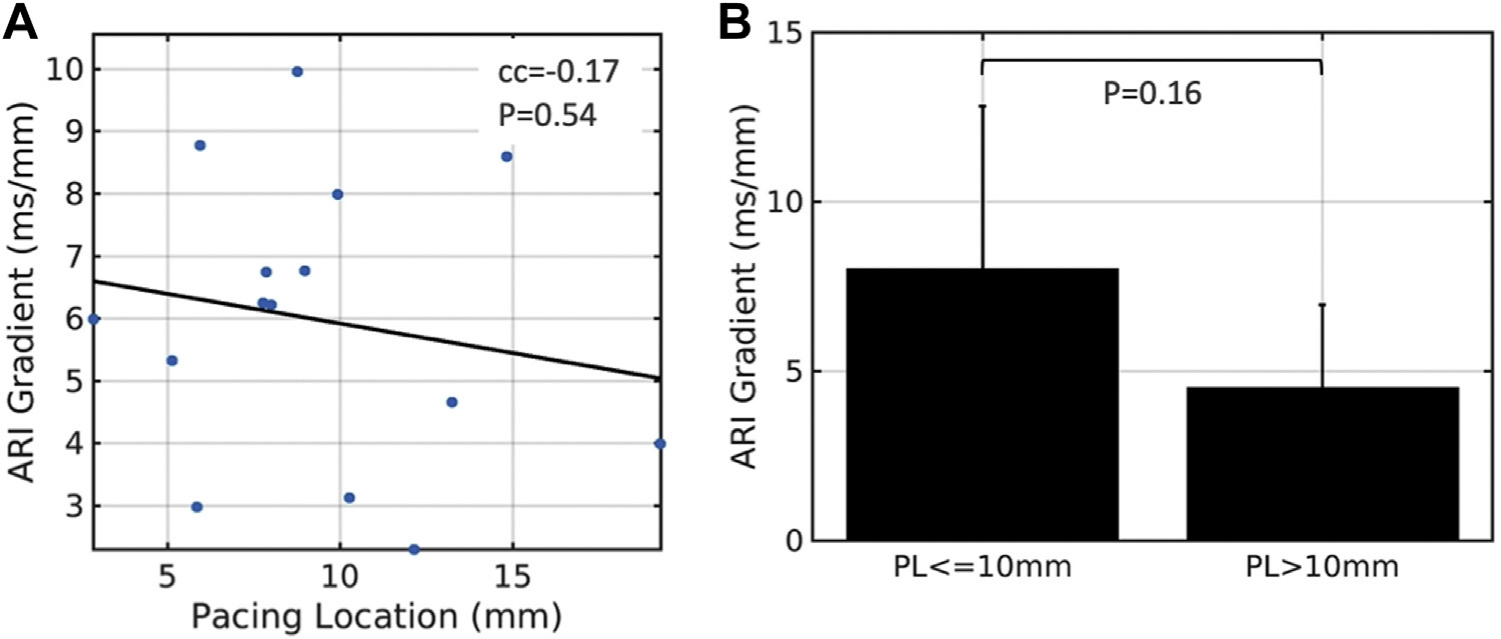
Effect of pacing distance from scar on activation recovery interval (ARI) gradients within areas of late gadolinium enhancement. **A:** Correlation between ARI gradient and pacing distance from scar. **B:** Comparison of ARI gradient between pacing location (PL) ≤10 mm vs >10 mm from scar. cc = correlation coefficient.

There was no significant correlation between mean ARI gradient within aLGE and pacing distance from the scar border (correlation coefficient –0.17; *P* = .54). There was no significant difference in ARI gradients when pacing in close proximity to (≤10 mm) vs distant (>10 mm) from scar (8.03 ± 4.79 mm/ms vs 4.53 ± 2.43 mm/ms; *P* = .16) (Figure 6). When repolarization time gradients within aLGE were calculated, there was also no correlation with pacing distance from scar (correlation coefficient 0.11; *P* = .68) and no difference between pacing ≤10 mm vs >10 mm from scar (*P* = .81) (Supplemental Figure 3).

## Discussion

The main findings from our study are as follows. (1) There were no detectable differences in mean ARI, ARI heterogeneity, or local ARI gradients between aLGE and healthy myocardium when assessed during pacing at multiple cycle lengths. (2) There were no significant differences in mean ARI, ARI heterogeneity, or local ARI gradients in either aLGE or healthy myocardium when RV and LV pacing were compared. (3) There was no significant difference in ARI gradients within aLGE when pacing in close proximity to vs distant from scar. (4) When repolarization time gradients were calculated instead of ARI gradients, there were no differences observed between aLGE and healthy myocardium or between RV and LV pacing, and there was similarly no effect of pacing distance from scar.

### Comparison of ARI and local ARI gradients between aLGE and healthy myocardium

We found no significant difference in mean ARI or ARI heterogeneity between aLGE and healthy myocardium. Action potential duration has been shown to change during ischemia and maturation of infarction. In a canine infarct study, action potential duration shortened acutely after infarction and then gradually lengthened over the subsequent 2–18 months, although measurements in the chronic phase were not significantly longer than baseline.^28^ A multitude of similar canine studies over the following 2 decades found similar results^15^; however, this infarct model generally involved *ex vivo* analysis of subepicardial tissue only. Moreover, these models are limited by significantly higher levels of collateral blood flow and a higher degree of repolarization variability in dogs compared to humans,^29^ thus highlighting the need for a more robust animal infarct model.

Studies of action potential duration in chronic infarct have shown conflicting results. This is likely related, at least in part, to the duration between the infarct and the data acquisition. A recent review of both human and animal studies found that the electrophysiological and structural properties of border zone varied greatly from 3 days to 12 months after infarct. The early phase was characterized by ionic changes and action potential duration shortening, and the chronic phase (>5 weeks) was characterized by structural (fibrotic) remodeling, with no difference in action potential duration between border zone and normal myocardium, independent of species studied.^15^ This is in keeping with the findings from our study.

More recently, in a human study of patients undergoing VT ablation, ARI was shown to be significantly longer in scar compared to border zone and normal myocardium.^14^ There are several possible reasons for the different findings between that study and our animal model study. First, the human study involved a small heterogeneous group of patients with a variety of cardiomyopathy etiologies, including ischemic and nonischemic, with different scar patterns, including endocardial and epicardial. Moreover, the patients had established cardiomyopathies; therefore, scar had matured over many years with different durations among patients. This differs from our animal model study in which scar was only ischemic, and infarct age and location were controlled between cases. Second, in the human study, electrograms were recorded from only a small number of sites, using a decapolar catheter placed across the border between normal myocardium and a pre-identified region of scar. In contrast, in our study, high-density mapping was performed across the entire LV, allowing acquisition of a large number of datapoints, including sites remote to the regions of scar. Third, in established cardiomyopathies, "normal" myocardium is likely to have undergone significant structural and ionic remodeling known to occur in heart failure,^30^ which may differ from the healthy myocardium defined in our infarct model.

Local heterogeneities in activation and repolarization times are likely to be more important than global differences between scar and normal myocardium in creating the substrate for reentry, and have been spatially correlated with VT sites of origin.^10^^,^^11^ Therefore, we compared local gradients of ARI between aLGE and normal myocardium but did not find a significant difference. Furthermore, it could be argued that the substrate for reentry is more dependent on absolute repolarization time at a given site, rather than ARI. However, we also found no significant effect of tissue type or pacing location on local repolarization time gradients. It is possible that there are significant gradients present around scar that are smaller than the resolution of the mapping technique, or are intramural, and thus are not detected in our model, although the use of unipolar electrograms should provide at least some intramural information. Therefore, our results suggest that high repolarization gradients are not an intrinsic property of all surviving myocytes within scar, and thus the substrate for reentry is more likely to be dependent on changes in tissue architecture and conduction velocity. Furthermore, it is important to note that complex neurohormonal interactions, which are known to be involved in arrhythmogenesis,^31^ may result in changes in ARI and local gradients that are not observed during a controlled study environment in which the subject typically is anesthetized.

### Comparison of ARI and local ARI gradients between RV pacing and LV endocardial pacing

When we compared RV pacing vs LV pacing, we found no significant differences in mean ARI, ARI heterogeneity, or ARI gradients within aLGE or healthy myocardium. Differences in repolarization between RV and LV endocardial pacing have not been well studied. During chronic endocardial RV pacing, activation of the LV occurs via slow conduction across the septum, resulting in an activation pattern similar to that of left bundle branch block. Engagement of the conduction system, via retrograde activation of the right bundle branch, may also contribute to LV activation, particularly if a lead is placed on the RV septum. In a porcine pacing study using noncontact mapping, RV apical pacing induced higher global distribution of ARI within the LV, compared to RV septal pacing.^32^ In a canine pacing model, LV apical pacing was associated with a higher apical to basal dispersion of repolarization compared to RV pacing.^33^ Results from our study do not support differences in repolarization between RV and LV endocardial pacing.

### Effect of endocardial pacing distance from scar on ARI gradients

We did not find a significant difference in ARI gradients within aLGE when pacing endocardially in close proximity to (≤10 mm) scar compared to distant from (>10 mm) scar. Previous computational modeling studies from our group demonstrated high repolarization gradients during LV epicardial pacing in close proximity to scar,^17^^,^^34^ and this may play a role in the development of ventricular arrhythmias that have been reported clinically during CRT.^34^ However, these high gradients were not observed during LV endocardial pacing.^18^ Physiological transmural action potential duration gradients were postulated to be the underlying mechanism for the differences seen between epicardial and endocardial LV pacing. Our findings are in keeping with these studies and support the hypothesis that, when pacing in proximity to scar, endocardial pacing may be less arrhythmogenic than epicardial pacing.

### Study limitations

Our study was limited by a small sample size of 6 pigs, although this is comparable to other animal model mechanistic studies, and the large number of datapoints collected during contact mapping provides confidence that the results in these subjects are reliable. Tissue staining and assessment of LV volumes and systolic function were not performed to confirm the presence of chronic infarct and myocardial remodeling; however, these have previously been demonstrated and validated for this pig infarct model.^20^^,^^22^ Myocardial scar was defined using LGE-CMR, which is the gold standard for scar imaging but may differ from electrophysiological definitions of scar using electrogram voltage.^35^ This definition was chosen because of the variable correlation between LGE-CMR and bipolar voltage maps as previously discussed. Registration of the LGE-CMR imaging and the anatomic shell obtained during electroanatomic mapping inevitably introduces a registration error; however, we excluded datapoints within 2 mm of the scar border to minimize any potential coregistration error. There may be some minor movement of the mapping catheter during cardiac contraction (between activation and repolarization times), which may affect the accuracy of ARI as a surrogate for action potential duration. However, ARI has been well validated in previous studies, with a very strong correlation between ARI and directly measured action potential duration in *in vivo* models during pacing.^36^ Furthermore, we also calculated local gradients using absolute repolarization times, rather than ARI, which would be less susceptible to the effects of contraction-related catheter movement, and found similar results compared to ARI gradients. Electrograms obtained in aLGE were characteristically heavily fractionated, making accurate calculations of activation and repolarization times difficult. To minimize error from inaccurate timings, points where ARI significantly differed between consecutive beats were excluded. Filtering reduced the number of included datapoints from aLGE, which could introduce type 2 error, making it difficult to detect true differences between aLGE and healthy tissue. The maximum pacing distance from scar in the study was 2 cm; therefore, we cannot exclude the possibility of significant differences in ARI gradients when pacing the ventricular myocardium at locations further from the scar border.

## Conclusion

In this porcine myocardial infarct model, we did not identify any significant differences in mean ARI, ARI heterogeneity, or ARI gradients between aLGE and myocardium, or between RV and LV endocardial pacing. Our findings suggest that global changes in ARI or local ARI gradients are not an intrinsic property of surviving myocytes within scar, and thus the substrate for reentry is more likely to be dependent on changes in conduction velocity and tissue architecture and on the dynamic effects of various complex neurohormonal interactions. We did not detect a significant effect of pacing distance from scar on ARI gradients. This supports previous computational modeling studies, which showed that, in contrast to LV epicardial pacing, LV endocardial pacing near scar does not affect dispersion of repolarization, and this may have implications for CRT delivery in patients with ischemic cardiomyopathy.
