# Supplementary material for: Effect of scar and pacing location on repolarization in a porcine myocardial infarction model
Source: Heart Rhythm O2. 2022 Jan 26;3(2):186–95. doi: 10.1016/j.hroo.2022.01.008 (PMC9043407; doi:10.1016/j.hroo.2022.01.008)
Supplement: Supplemental Tables 1 and 2 [file mmc1.docx]

**Supplementary Figures:**

**
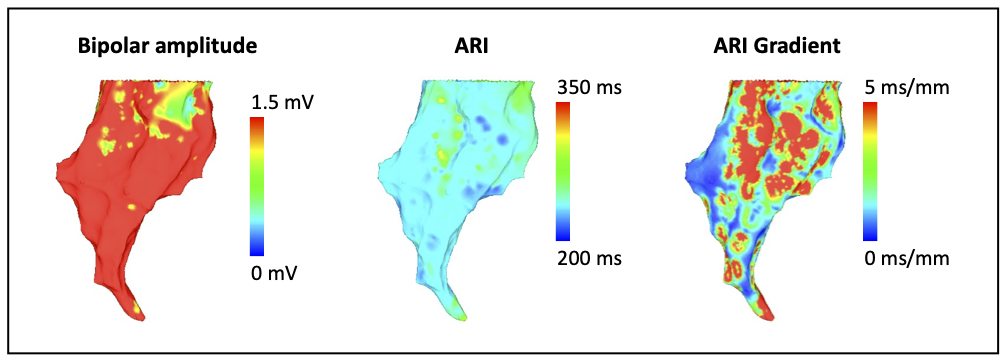
**

**Supplementary Figure 1**. Example maps from sham control pig. Bipolar amplitude map (left), activation recovery interval (ARI) map (middle) and ARI gradient map (right). All maps are during right ventricular pacing.


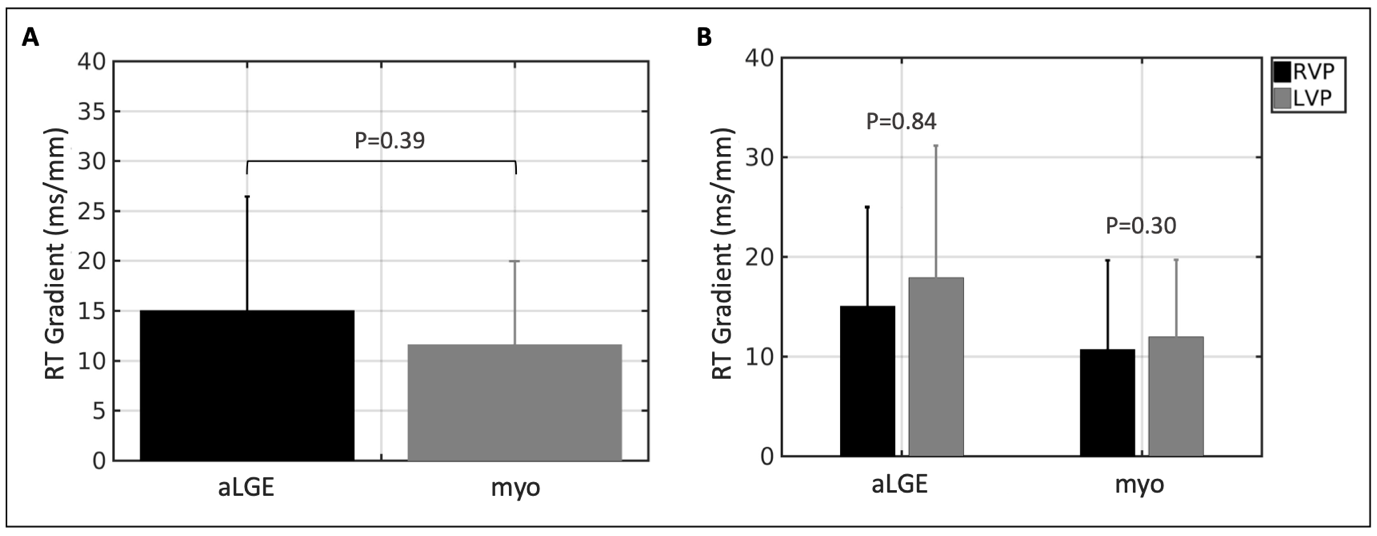


**Supplementary Figure 2**. Repolarization time (RT) gradients.

(A) Mean RT gradients in areas of late gadolinium enhancement (aLGE) vs healthy myocardium (myo). (B) Mean RT gradient within aLGE and myo during right ventricular pacing (RVP) and left ventricular endocardial pacing (LVP).

**
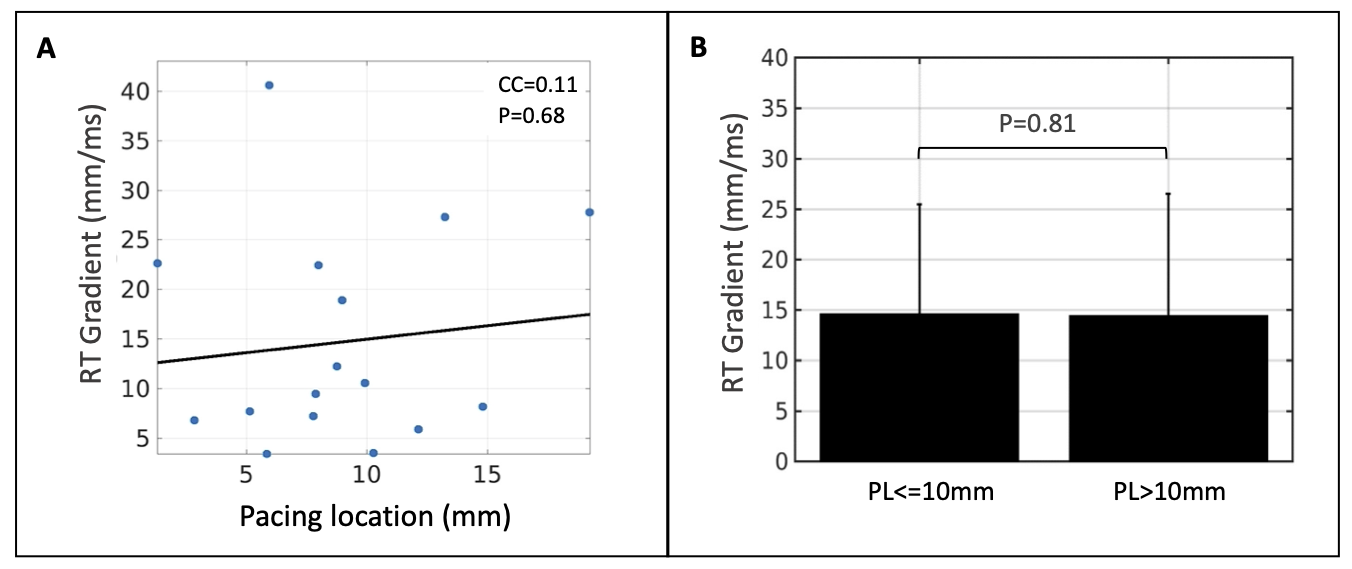
**

**Supplementary Figure 3**. Effect of pacing distance from scar on repolarization time (RT) gradients within areas of late gadolinium enhancement (aLGE).

(A) Correlation between RT gradient and pacing distance from scar. (B) Comparison of RT gradient between pacing location (PL) ≤10 mm vs >10 mm from scar. cc = correlation co-efficient.
