# Supplementary material for: Effect of scar and pacing location on repolarization in a porcine myocardial infarction model
Source: Heart Rhythm O2. 2022 Jan 26;3(2):186–95. doi: 10.1016/j.hroo.2022.01.008 (PMC9043407; doi:10.1016/j.hroo.2022.01.008)
Supplement: Supplemental Figure 1-3 [file mmc2.docx]

| **Comparison** | **PCL (ms)** | **Mean ± S.D. (ms)** | **P-value** |
| --- | --- | --- | --- |
| **Mean ARI** | | | |
| aLGE vs myo | 300 | 181.15 ± 5.68 vs 182.06 ± 8.24 | 0.58 |
| aLGE vs myo | 500 | 302.78 ± 18.29 vs 299.66 ± 19.21 | 0.46 |
| RVP vs LVP (aLGE) | 300 | 178.14 ± 5.79 vs 181.13 ± 4.42 | 0.54 |
| RVP vs LVP (aLGE) | 500 | 311.21 ± 25.78 vs 291.21 ± 3.28 | 0.23 |
| RVP vs LVP (myo) | 300 | 176.97 ± 6.15 vs 190.98 ± 8.05 | 0.14 |
| RVP vs LVP (myo) | 500 | 301.29 ± 27.78 vs 295.05 ± 15.29 | 0.49 |
| **ARI heterogeneity** | | | |
| aLGE vs myo | 300 | 13.60 ± 4.86 vs 16.13 ± 6.30 | 0.29 |
| aLGE vs myo | 500 | 23.51 ± 11.65 vs 24.62 ± 12.89 | 0.66 |
| RVP vs LVP (aLGE) | 300 | 13.22 ± 4.76 ± 14.19 ± 6.00 | 0.80 |
| RVP vs LVP (aLGE) | 500 | 23.97 ± 9.69 vs 26.65 ± 14.53 | 0.83 |
| RVP vs LVP (myo) | 300 | 20.99 ± 7.79 vs 13.41 ± 2.17 | 0.12 |
| RVP vs LVP (myo) | 500 | 21.54 ± 6.51 vs 28.44 ± 19.25 | 0.58 |

**Supplementary Table 1**. Activation recovery interval (ARI) data for second exported beat. aLGE: areas of late gadolinium enhancement; LVP: left ventricular pacing; myo: healthy myocardium; PCL: pacing cycle length; S.D.: standard deviation; RVP: right ventricular pacing

| **Comparison** | **PCL (ms)** | **Mean ± S.D. (ms)** | **P-value** |
| --- | --- | --- | --- |
| **Mean ARI** | | | |
| aLGE vs myo | 300 | 181.26 ± 5.79 vs 185.97 ± 10.03 | 0.10 |
| aLGE vs myo | 500 | 303.49 ± 18.84 vs 300.13 ± 19.21 | 0.44 |
| RVP vs LVP (aLGE) | 300 | 178.17 ± 6.04 vs 181.31 ± 4.44 | 0.52 |
| RVP vs LVP (aLGE) | 500 | 311.76 ± 27.14 vs 292.20 ± 3.38 | 0.27 |
| RVP vs LVP (myo) | 300 | 176.89 ± 6.17 vs 191.01 ± 7.96 | 0.13 |
| RVP vs LVP (myo) | 500 | 301.60 ± 27.56 ± 295.53 ± 15.33 | 0.50 |
| **ARI heterogeneity** | | | |
| aLGE vs myo | 300 | 13.69 ± 4.78 vs 16.23 ± 6.34 | 0.29 |
| aLGE vs myo | 500 | 23.41 ± 11.51 vs 24.74 ± 12.94 | 0.60 |
| RVP vs LVP (aLGE) | 300 | 13.30 ± 4.69 vs 14.34 ± 5.95 | 0.79 |
| RVP vs LVP (aLGE) | 500 | 23.22 ± 9.10 vs 26.95 ± 14.76 | 0.76 |
| RVP vs LVP (myo) | 300 | 21.09 ± 7.87 vs 13.47 ± 2.31 | 0.12 |
| RVP vs LVP (myo) | 500 | 21.61 ± 6.50 vs 28.62 ± 19.42 | 0.58 |

**Supplementary Table 2**. Activation recovery interval (ARI) data for average of both exported beats. aLGE: areas of late gadolinium enhancement; LVP: left ventricular pacing; myo: healthy myocardium; PCL: pacing cycle length; S.D.: standard deviation; RVP: right ventricular pacing
